# Zfra activates memory Hyal-2+ CD3− CD19− spleen cells to block cancer growth, stemness, and metastasis *in vivo*

**DOI:** 10.18632/oncotarget.2895

**Published:** 2015-02-19

**Authors:** Ming-Hui Lee, Wan-Pei Su, Wan-Jen Wang, Sing-Ru Lin, Chen-Yu Lu, Yu-An Chen, Jean-Yun Chang, Shenq-Shyang Huang, Pei-Yi Chou, Siou-Ru Ye, Szu-Jung Chen, Huan He, Ting-Hsiu Liu, Ying-Tsen Chou, Li-Jin Hsu, Feng-Jie Lai, Shean-Jen Chen, Hoong-Chien Lee, David Kakhniashvili, Steven R. Goodman, Nan-Shan Chang

**Affiliations:** ^1^ Institute of Molecular Medicine, National Cheng Kung University Medical College, Tainan, Taiwan, ROC; ^2^ Institute of Basic Medical Sciences, National Cheng Kung University Medical College, Tainan, Taiwan, ROC; ^3^ Department of Medical Laboratory Science and Biotechnology, National Cheng Kung University Medical College, Tainan, Taiwan, ROC; ^4^ Center of Infectious Disease and Signal Research, National Cheng Kung University, Tainan, Taiwan, ROC; ^5^ Department of Dermatology, Chi-Mei Medical Center, Tainan, Taiwan, ROC; ^6^ Advanced Optoelectronic Technology Center, National Cheng Kung University Medical College, Tainan, Taiwan, ROC; ^7^ Graduate Institute of Systems Biology and Bioinformatics, National Central University, Zhongli, Taiwan, ROC; ^8^ Institute of Biomedical Sciences and Technology, Department of Biochemistry and Molecular Biology, SUNY Upstate Medical University, Syracuse, NY, USA; ^9^ Department of Neuroscience and Physiology, SUNY Upstate Medical University, Syracuse, NY, USA

**Keywords:** Zfra, skin cancer, melanoma, metastasis, stemness

## Abstract

Zfra is a 31-amino-acid zinc finger-like protein, which participates in the tumor necrosis factor signaling. Here, we determined that when nude mice and BALB/c mice were pre-injected with nanogram levels of a synthetic Zfra1–31 or truncated Zfra4–10 peptide via tail veins, these mice became resistant to the growth, metastasis and stemness of melanoma cells, and many malignant cancer cells. The synthetic peptides underwent self-polymerization in phosphate-buffered saline. Alteration of the Ser8 phosphorylation site to Gly8 abolished Zfra aggregation and its-mediated cancer suppression *in vivo*. Injected Zfra peptide autofluoresced due to polymerization and was trapped mainly in the spleen. Transfer of Zfra-stimulated spleen cells to naïve mice conferred resistance to cancer growth. Zfra-binding cells, designated Hyal-2+ CD3− CD19− Z cells, are approximately 25–30% in the normal spleen, but are significantly downregulated (near 0–3%) in tumor-growing mice. Zfra prevented the loss of Z cells caused by tumors. *In vitro* stimulation or education of naïve spleen cells with Zfra allowed generation of activated Z cells to confer a memory anticancer response in naïve or cancer-growing mice. In particular, Z cells are abundant in nude and NOD-SCID mice, and can be readily activated by Zfra to mount against cancer growth.

## INTRODUCTION

By functional cloning, we have previously isolated a 31-amino-acid Zfra (zinc finger-like protein that regulates apoptosis) [1–4]. This small protein possesses two cysteines and one histidine in the amino acid sequence, and is similar to C2H2 type zinc finger proteins [1–4]. Zfra participates in the tumor necrosis factor (TNF)-mediated apoptosis [1–5]. When transiently overexpressed *in vitro*, Zfra induces growth suppression and apoptosis of many types of cells probably by activating and interacting with adaptor proteins in the TNF signal pathway, including TNF receptor-associated death domain protein (TRADD), Fas-associated death domain protein (FADD), and receptor-interacting protein (RIP). Zfra also binds nuclear factor NF-κB, cJun *N*-terminal kinase 1 (JNK1), and tumor suppressors p53 and WW domain-containing oxidoreductase (WWOX, FOR, or WOX1) [1–4]. Probably due to its small size, Zfra is capable of interacting with both the *N*- and *C*-termini of WWOX [2, 6–10].

In response to UV light, the endogenous level of Zfra is upregulated [1–3]. Zfra becomes Ser8 phosphorylated and translocates mainly to the nuclei [3]. Interestingly, induction of the mitochondrial pathway of apoptosis by staurosporine or betulinic acid, Zfra becomes Ser8-phosphorylated and translocates to the mitochondria to induce apoptosis [3, 4]. Alteration of Ser8 abolishes relocation of Zfra to the mitochondria and no cell death occurs [3, 4]. Zfra exhibits an intriguing characteristic in inducing the mitochondrial pathway of apoptosis. We have determined that transiently overexpressed Zfra significantly downregulates Bcl-2 and Bcl-xL, blocks cytochrome c release from the mitochondria, and yet induces dissipation of mitochondrial membrane potential, thus leading to apoptosis [3, 4]. This novel finding runs against a general belief that suppression of apoptosis inhibitors Bcl-2 and Bcl-xL results in spontaneous cytochrome c release and causing apoptosis [11, 12]. One of the mechanisms for Zfra inhibition of cytochrome c release is that transiently overexpressed WWOX induces cytochrome c release, and that Zfra binds and blocks the function of WWOX [3]. In light of the *in vitro* findings, here we examined the functional properties of Zfra *in vivo*. We showed that pre-treatment of mice with synthetic Zfra peptides, either full-length or truncated, results in protection of mice against the growth of many types of cancers.

## RESULTS

### Synthetic Zfra peptide polymerizes in PBS and blocks skin cancer growth, metastasis, and stemness *in vivo*

**Figure 1:**
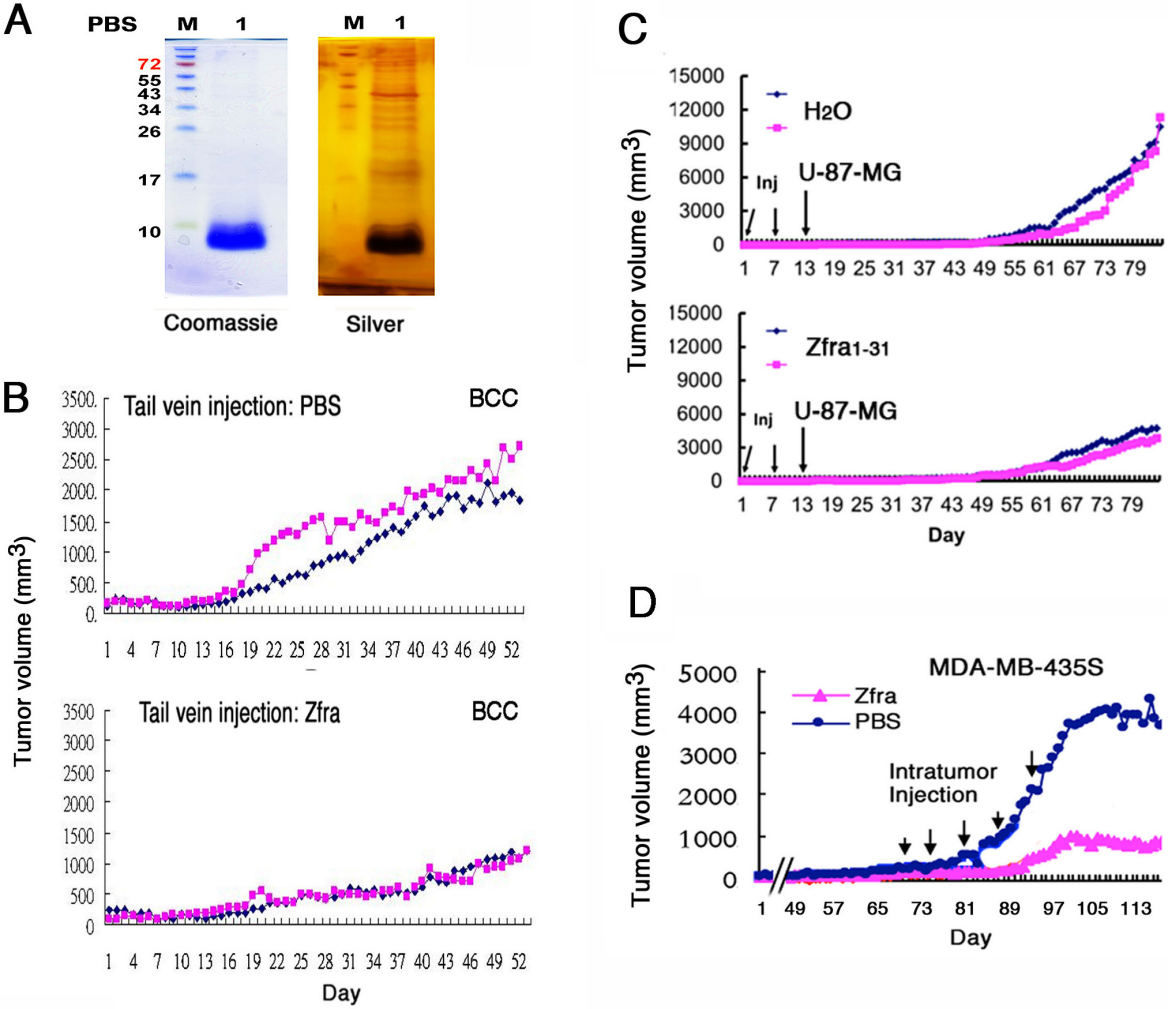
Zfra peptide self-polymerizes *in vitro* and blocks cancer growth *in vivo* **(A)** Synthetic full-length Zfra1–31 of 3.5 kDa (2 mM) polymerized in PBS (degassed) during incubation at room temperature for 16–24 hr. Silver staining of a reducing SDS-PAGE revealed the polymerized bands of greater than 80 kDa. **(B)** Nude mice were pre-injected with 100 μl Zfra1–31 (2 mM in PBS) or sterile PBS via tail veins once per week for 3 weeks, followed by inoculating with skin BCC cells (2 million cells /100 μl PBS) at both the right and left flanks. Tumor at left is marked in red, and tumor at right in blue. Tumor volumes were measured (cubic mm) daily. A represent data is shown from 3 experiments. In end-point experiments, tumor suppression by Zfra1–31 is 65 ± 17% (mean ± standard deviation from 6 tumors in controls and 6 in experiments). **(C)** Nude mice were pre-injected with sterile MilliQ water or Zfra1–31 (3 mM in 100 μl sterile water) in 2 or 3 consecutive weeks, followed by inoculating 2 million cells of glioma U-87-MG. **(D)** Direct injection of Zfra1–31 (2 mM in 100 μl PBS) to a solid breast tumor MDA-MB-435s resulted in suppression of the cancer growth.

The full-length Zfra1–31 peptide was synthesized. The sequence is: NH-MSSRRSSSCKYCEQDFRAHTQ KNAATPFLAN-COOH, where Ser8 is a known phosphorylation site [2, 3]. In MilliQ water, Zfra1–31 went into solution (at 1–10 mM) without visible precipitation. However, when Zfra peptide was suspended in degassed phosphate-buffered saline (PBS) at greater than 5 mM, Zfra went out of solution and became visible precipitates in less than 5 hr (Supplementary Figure S1). Similar results were observed by dissolving Zfra peptide in dimethyl sulfoxide (DMSO). By reducing SDS-PAGE, monomeric Zfra of 3.5 kDa was shown to polymerize up to 80 kDa and higher in a stepwise manner, as stained by Commassie blue and silver stain (Figure 1A).

We examined whether Zfra exerts anticancer activity *in vivo*. Many types of malignant or metastatic cancer cells were chosen to address whether Zfra suppresses cancer growth, stemness, and metastasis. In general, breast, brain, skin, and lung cancer cells were mainly used in this study. T cell-deficient nude mice were pre-injected with 100 μl of the full-length Zfra1–31 at 2 mM in sterile PBS or PBS only via tail veins once per week for 3 weeks. These mice were allowed to take a rest for a week, and then inoculated with skin basal cell carcinoma (BCC) cells at both the right and left flanks. Compared to controls, Zfra blocked BCC growth by greater than 50% (Figure 1B). In end-point experiments, tumor suppression by Zfra1–31 is 65 ± 17% (*n* = 6). All synthetic peptides for this and the following experiments were prepared in degassed sterile MilliQ water as 10 mM stocks and kept in a –80^o^C freezer. Diluted peptides in degassed sterile MilliQ water or PBS were freshly prepared for tail vein injections.

Similarly, pre-injection of nude mice with Zfra1–31 twice via tail veins, followed by inoculating with malignant glioblastoma U-87-MG cells a week later, resulted in suppression or prevention of cancer growth (Figure 1C or Supplementary Figure S2A). However, Zfra1–31 did not suppress the growth of glioblastoma 13–06-MG cells in nude mice (Supplementary Figure S2B). Direct intratumor injection of Zfra1–31 to a solid malignant breast cancer MDA-MB-435s suppressed the cancer growth by greater than 80% (Figure 1C). The observations suggest that the T-cell defective immune system in nude mice can be activated or charged by Zfra for the anticancer response.

**Figure 2:**
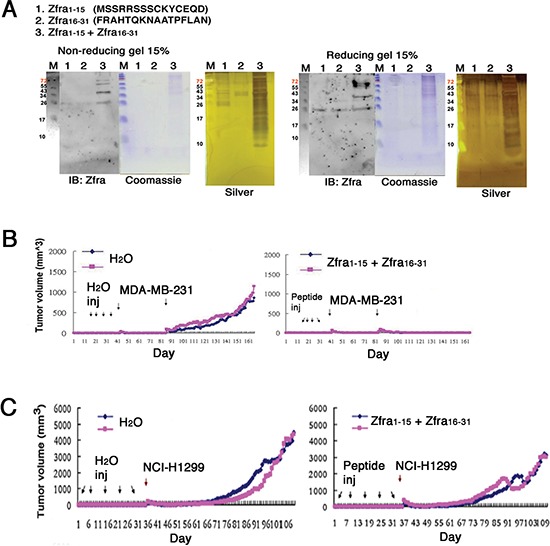
Zfra1–15 and Zfra16–31 peptide mixture polymerizes *in vitro* and blocks cancer growth *in vivo* **(A)** Synthetic Zfra1–15 and Zfra16–31 were incubated alone (2 mM in 100 μl PBS for lanes 1 and 2) or mixed together (lane 3) at room temperature for 24 hr. Dramatic polymerization of the peptide mixture occurred, as shown in both non-reducing and reducing gels. Less polymerization occurred when the peptides were resuspended in MilliQ water. **(B)** Pre-injection of nude mice with the Zfra1–15 / Zfra16–31 mixtures (in sterile MilliQ water) resulted in blocking of the growth of breast MDA-MB-231 cells. **(C)** Under similar conditions, the peptide mixtures were less effective in blocking the growth of lung NCI-H1299 cells.

To elucidate how Zfra undergoes self-polymerization, Zfra peptides of amino acid #1–15 (Zfra1–15) and #16–31 (Zfra16–31) were made. The peptides were dissolved in sterile MilliQ water and incubated alone at room temperature for 48 hr. Low levels of polymerization were shown in these peptides (see lanes 1 and 2 in Figure 2A). When both peptides were mixed and incubated together, dramatic polymerization of Zfra1–15 with Zfra16–31 occurred in a ladder-like pattern under both reducing and non-reducing conditions (lane 3 in Figure 2A). The observations suggest that Zfra1–15 and Zfra16–31 directly interact with each other. Since reducing conditions did not break apart the polymers, specific amino acid residues, other than cysteines (e.g. Cys9 and Cys12), in both peptides are involved in the polymerization. When Zfra1–15 and Zfra16–31 were mixed in sterile MilliQ water and then delivered in nude mice via tail veins, effective blocking of malignant breast MDA-MB-231 cell growth occurred (Figure 2B). Similar results were observed when both peptides were suspended in PBS (data not shown). Under similar conditions, the peptide mixtures in sterile MilliQ water were less effective in blocking of the growth of lung NCI-H1299 cells (Figure 2C).

### Self-polymerization of a 7-amino-acid Zfra4–10 is sufficient in blocking cancer growth

**Figure 3:**
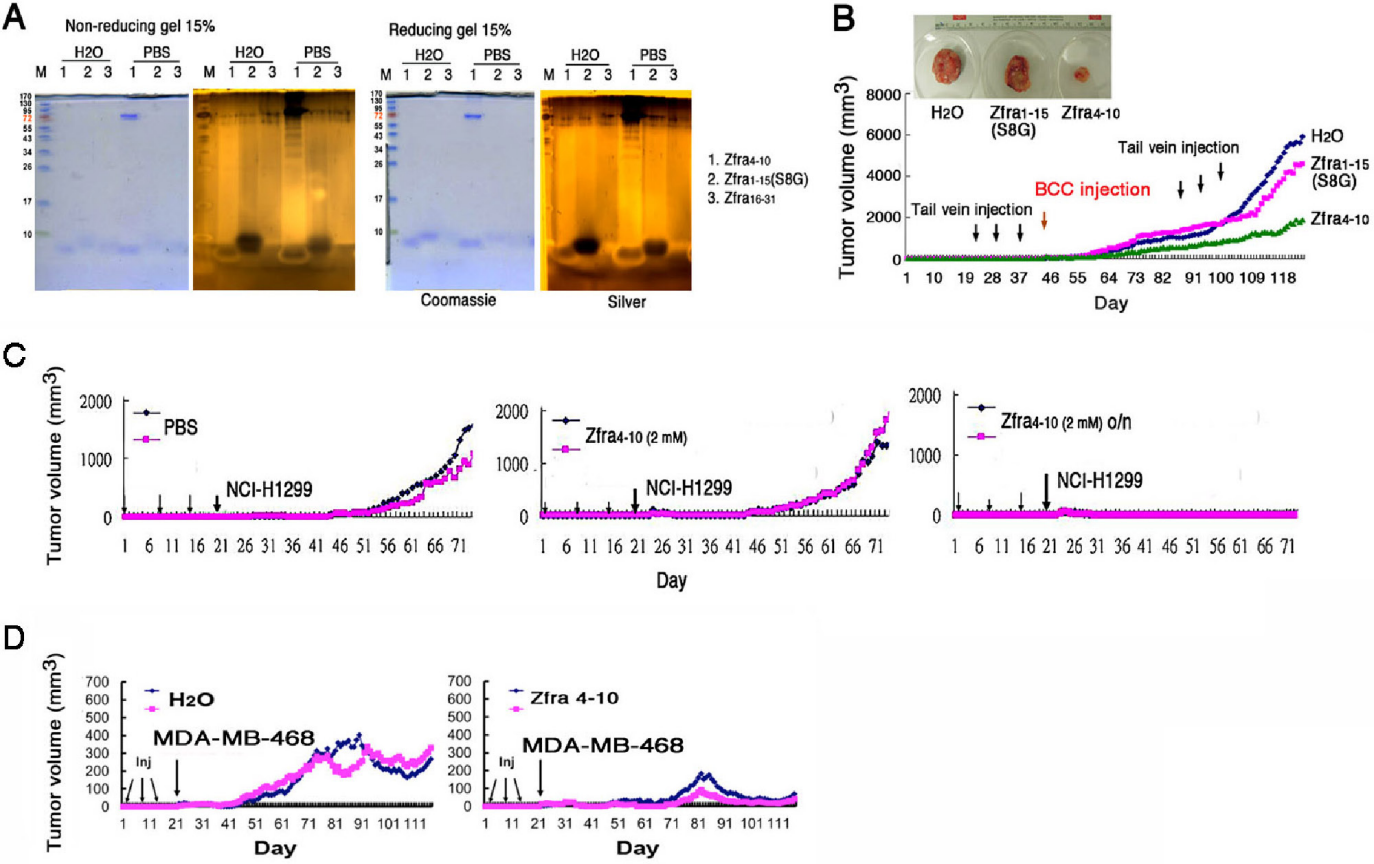
Zfra4–10 polymerization and correlation with its anticancer activity **(A)** Zfra4–10 (2 mM) underwent self-polymerization effectively in PBS during incubation at room temperature for 24 hr. Alteration of Ser8 to Gly8 abolished the self-polymerization in Zfra1–15. **(B)** Nude mice were pre-injected with Zfra1–15(S8G) or Zfra4–10 (1 mM in 100 μl sterile MilliQ water), followed by BCC inoculation. Boost injection for Zfra was carried out 2 months later. Zfra4–10 suppressed tumor growth. S8G mutant was not effective. Control mice were treated with sterile MilliQ water only. A representative data from an average of 2 tumors for each experiment is shown. In end-point experiments, B16F10 tumor suppression by Zfra4–10 is 85 ± 12% (mean ± standard deviation from 12 tumors in controls and 12 tumors in experiments). Similar results were observed when Zfra4–10 was suspended in sterile PBS and used for experiments. **(C)** Zfra4–10 peptides were freshly resuspended in 100 μl sterile PBS, or the preparations incubated overnight (o/n), prior to tail vein injections to nude mice. Inoculation with lung NCI-H1299 cells was carried out one week later. Tumor sizes were measured daily. **(D)** Pre-treatment of nude mice with Zfra4–10 (3 mM in sterile MilliQ water) blocked the growth of inoculated breast MDA-MB-468 cells.

We investigated the shortest functional segment(s) in Zfra to block cancer growth *in vivo*. By sequential deletion and functional testing *in vivo*, a synthetic 7-amino-acid Zfra4–10 peptide (RRSSSCK) was determined to be functionally active in suppressing skin cancer growth. Zfra4–10 effectively underwent ladder-like polymerization in PBS but much less effective in water (Figure 3A). This peptide is within the sequence of Zfra1–15, and Zfra1–15 also underwent partial ladder-like polymerization (Figure 2A) and blocked cancer growth (data not shown). Alteration of Ser8 to Gly8 in Zfra1–15(S8G) abolished its polymerization in PBS (Figure 3A). Again, Zfra16–31 was less effective (Figures 2A and 3A).

Nude mice received the Zfra4–10 peptide or sterile MilliQ water via tail veins, followed by resting for a week and then inoculating with BCC (Figure 3B). Compared with control mice, Zfra4–10-treated mice resisted the growth of BCC by greater than 75% suppression (Figure 3B). Zfra1–15 exerted a similar extent of cancer growth suppression (data not shown). Alteration of Ser8 to Gly8 in Zfra1–15 abolished its activity in suppressing cancer growth (Figure 3B). Secondary booster injections with peptides at 70 days later did not result in increased suppression of BCC growth (Figure 3B).

We examined the efficacy of Zfra4–10 in self-polymerization and correlation with its anticancer activity. Zfra4–10 peptide was freshly prepared in sterile PBS, followed by immediate tail vein injection in nude mice. Or, the peptide was incubated overnight at room temperature prior to tail vein injections. At 2 mM, the freshly prepared Zfra4–10 did not confer cancer suppression against the growth of NCI-H1299 cells (Figure 3C). However, the overnight incubated Zfra4–10, which had undergone further polymerization, provided a complete suppression (Figure 3C). At higher concentrations (> 2mM), overly polymerized or aggregated Zfra4–10 failed to exert its anticancer effect (data not shown). However, preparation of Zfra4–10 in sterile MilliQ water was sufficient to block the growth of breast MDA-MB-468 cells (Figure 3D), suggesting that depending upon cancer cell types, Zfra4–10 may act differently in blocking their growth.

### Zfra4–10 suppresses melanoma growth, metastasis and stemness

**Figure 4:**
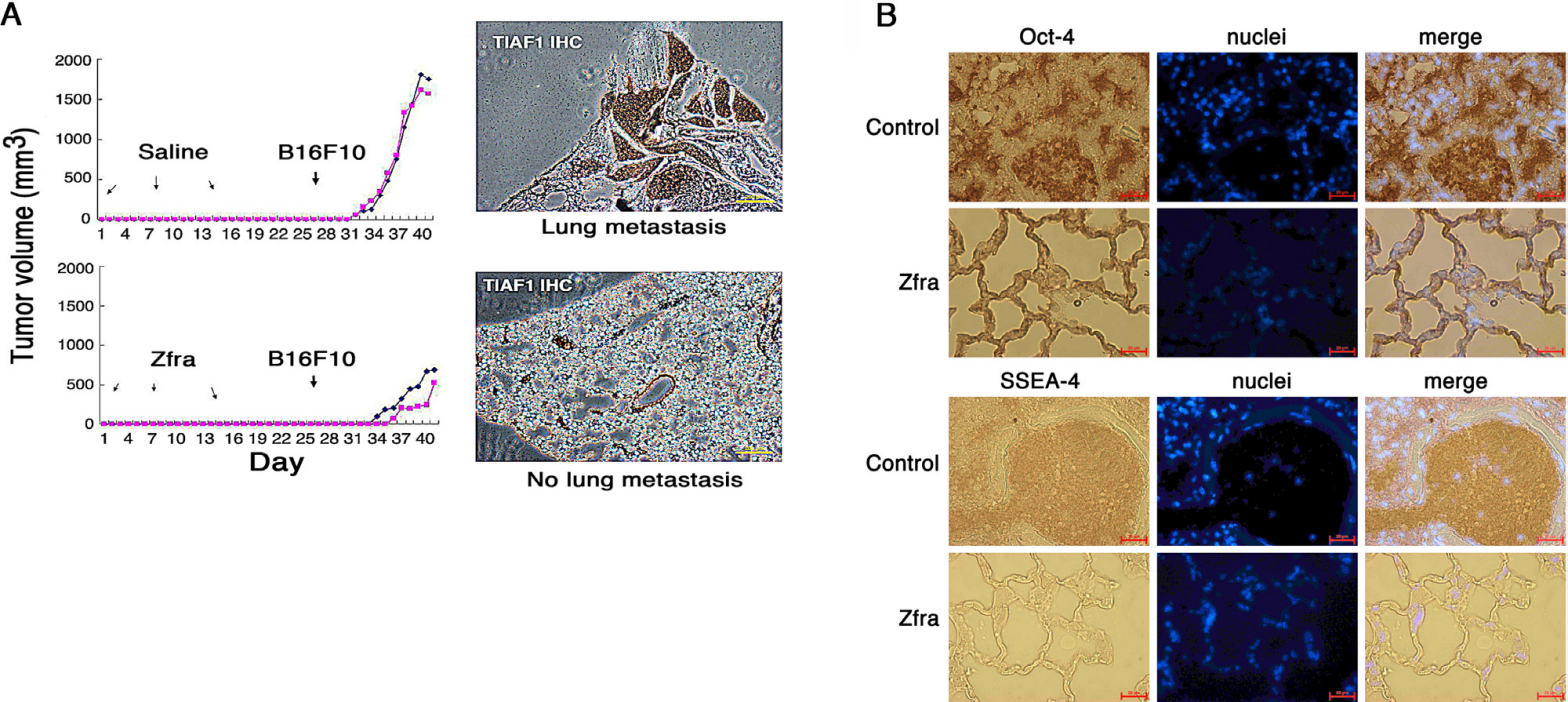
Zfra4–10 blocks skin cancer growth *in vivo* **(A)** Nude mice were pre-injected with 100 μl of sterile MilliQ water or Zfra4–10 (2 mM in sterile water) for 3 consecutive weeks, followed by inoculating with malignant melanoma B16F10 cells. Zfra4–10 inhibited B16F10 metastasis to the lung. TIAF1 is upregulated during cancer progression as determined by immunohistochemistry (IHC). **(B)** Zfra4–10 suppressed the expression of pluripotent stem cell markers Oct-4 and SSEA-4 in the lungs of nude mice.

Next, we examined whether Zfra regulates melanoma growth and metastasis. Nude mice were pre-injected with sterile MilliQ water or Zfra4–10 (2 mM in 100 μl sterile water) for 3 consecutive weeks, followed by resting for 2 weeks and then inoculating with malignant melanoma B16F10 cells (Figure 4A). Compared to controls, Zfra effectively blocked the growth of B16F10 and metastasis to the lung (Figure 4A). TGF-β-induced antiapoptotic factor (TIAF1), which is known to form fibrous materials in many types of cancer tissues [13–15], was upregulated in the lung of the control mice during cancer metastasis, as determined by immunohistochemistry (Figure 4A). The fibrous materials are associated with collagen and are considered to be protective for the growth of many types of cancer cells [13–15]. In contrast, monomeric TIAF1 acts synergistically with tumor suppressor p53 and WWOX to counteract cancer growth and migration, whereas both tumor suppressors may have loss-of-function or completely disappeared in metastatic cancer cells [14, 15]. In parallel experiments, Zfra suppressed the stemness of B16F10 cells in the lungs of nude mice by blocking the expression of pluripotent stem cell markers Oct-4 and SSEA-4 (Figure 4B). In control nude mice, expression of Oct-4 and SSEA-4 was observed (Figure 4B).

We examined whether Zfra works in immune competent BALB/c mice. These mice were received Zfra4–10 or sterile MilliQ water via tail vein injections in 3 consecutive days. Following resting for one week, these mice were injected with B16F10 cells via tail veins. B16F10 cells were shown to grow in the lung in the control mice, but not in the Zfra-treated mice (data not shown). The results are in parallel with the inhibition of B16F10 metastasis to the lung in the Zfra-treated nude mice (Figure 4A). Indeed, alterations of Zfra4–10 amino acid sequence abolished its self-polymerization and anticancer function. The alterations included deletion of Arg4 and Cys9 and/or adding a phosphate group to Ser8. That is, peptides used for these experiments were Zfra5–10, Zfra1–15(S8G), Zfra4–10(pS8), Zfra5–10(ΔC9), Zfra5–10(ΔC9/pS8), and WWOX28–42 (as a control peptide). Under similar conditions, none of these peptides underwent polymerizations and failed to block B16F10 growth in nude mice (data not shown).

### Zfra4–10 induces a memory anticancer response against many types of cancer cells *in vivo*

**Figure 5:**
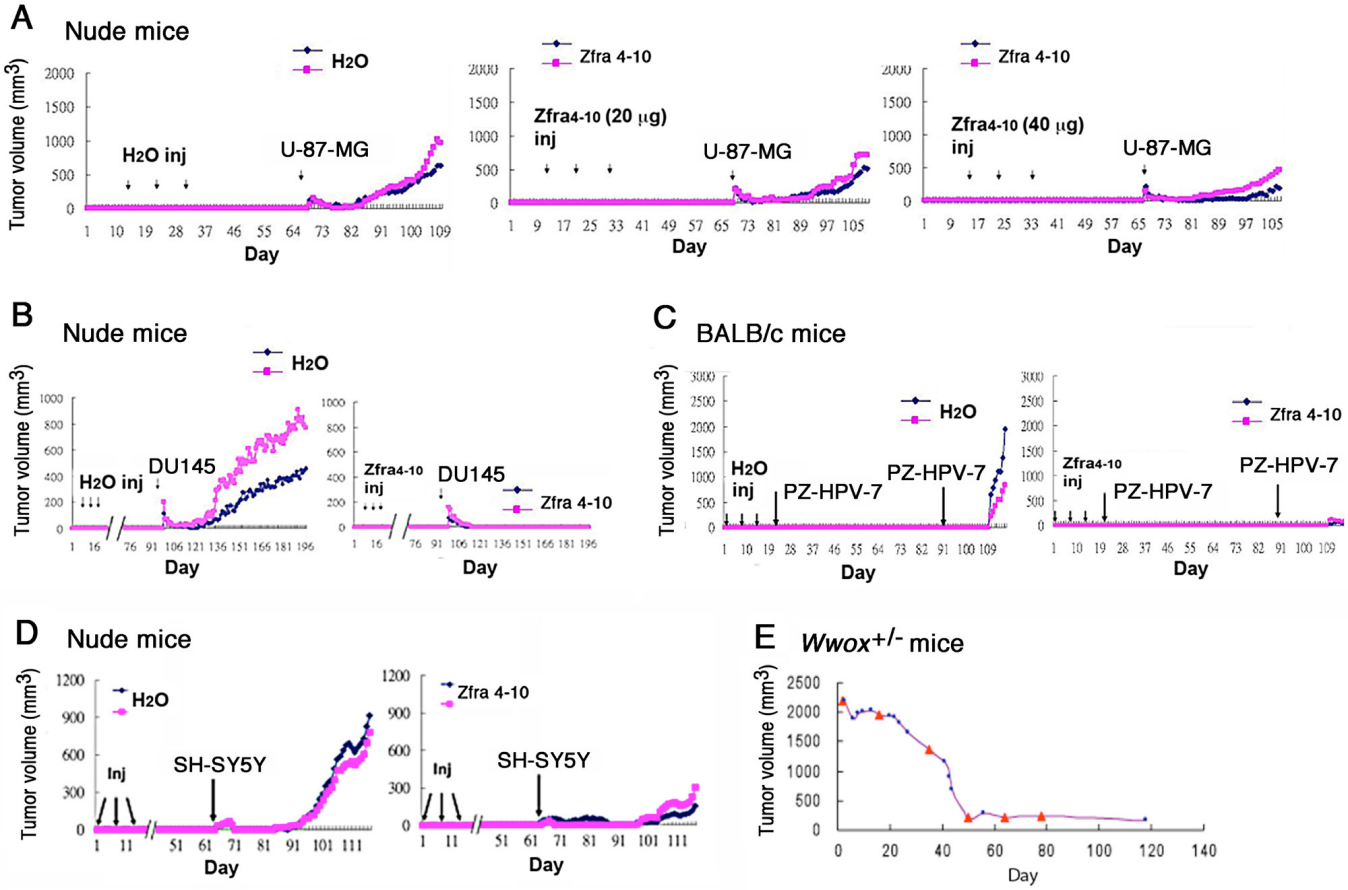
Zfra induces a memory anticancer response against a broad spectrum of cancer cells **(A)** Nude mice received Zfra4–10 (2 and 4 mM, or 10 and 20 μg, in 100 μl sterile MilliQ water) or sterile water via tail veins once per week for 3 weeks. One month later, the mice were inoculated with glioma U-87-MG cells. **(B)** Nude mice received Zfra4–10 (2 mM in 100 μl sterile water) or sterile MilliQ water via tail veins once per week for 4 weeks. Three months later, these mice were inoculated with malignant prostate cancer DU145 cells. **(C)** BALB/c mice were pre-injected with sterile MilliQ water or Zfra4–10 (2 mM in 100 μl sterile water) in 3 consecutive weeks, followed by inoculating non-invasive prostate PZ-HPV-7 cancer cells 7 and 70 days later, respectively. Tumor sizes were measured daily. **(D)** Nude mice were pre-injected with sterile MilliQ water or Zfra4–10 (3 mM in 100 μl sterile water) in 3 consecutive weeks. Two months later, mice were inoculated with neuroblastoma SH-SY5Y cells at 2 subcutaneous sites on both flanks. For above experiments **(A–D)**, a representative data set is shown from 2–4 experiments. **(E)** Shown is a heterozygous *Wwox*+/− mouse with exon 2–4 ablation and spontaneously developing tumor in the abdominal area. Via tail vein injection (see red triangles), Zfra4–10 (2 mM in 100 μl sterile water) completely cured the tumor in 50 days. Ten mice were examined and the cure rate was 75%.

We examined a broad spectrum of cancer cell lines and determined whether Zfra4–10 blocks the cancer growth *in vivo*, and showed that Zfra4–10 induced a memory anticancer response. T cell-deficient nude mice were pretreated with Zfra4–10. A month later, these mice were inoculated with malignant glioma U-87-MG cells on both flanks. Zfra-treated mice resisted the growth of U-87-MG cells (40–50% inhibition; Figure 5A). The one-month resting period did not result in reduction of the efficacy of Zfra in suppressing cancer growth.

Additionally, nude mice were challenged with Zfra4–10 and then inoculated with malignant prostate cancer DU145 cells 3 months later. The results showed that Zfra4–10 completely blocked the growth of the implanted DU145 cells in nude mice (100% inhibition; Figure 5B), suggesting that Zfra-activated anticancer event is memorized in the mice. Under similar conditions, immune competent BALB/c mice were pretreated with Zfra4–10 or sterile water 3 times, and then inoculated with prostate cancer PZ-HPV-7 cells, respectively, 1 week and 3 months later. Zfra4–10 completely blocked the growth of PZ-HPV-7 cells (100% inhibition; Figure 5C). PZ-HPV-7 is a non-invasive, virus-transformed epithelial cells derived from the peripheral zone of the prostate [16].

Also, Zfra4–10 effectively suppressed the growth of neuroblastoma SH-SY5Y cells (>70% inhibition; Figure 5D). There was a 3-month resting period between Zfra4–10 injection and SH-SY5Y inoculation, further attesting that Zfra-induced anticancer activity is memorized in the T cell-deficient nude mice. We have developed knockout mice for tumor suppressor gene *Wwox* [17, 18]. Heterozygous *Wwox*+/− appeared to spontaneously develop tumors around 9 months old. Zfra completely cured the cancer in 50 days, and the successful rate was 70% by examining 10 mice (Figure 5E).

Finally, malignant glioblastoma cells showed different susceptibility to Zfra. In agreement with the aforementioned observations, both Zfra1–31 and Zfra4–10 effectively prevented the growth of glioma U-87-MG cells (~60% inhibition; Figures 1C, 5A and Supplementary S2A), but had no inhibitory effects on 13–06-MG and CNS-1 glioma cells (Figure S2B, S2C). In similar experiments, Zfra1–15 and Zfra16–31 in combination did not block the growth of glioma 13–06-MG cell growth but effectively prevented cancer metastasis to the lung (Supplementary Figure S3A, S3B). This is in agreement with the observation for Zfra complete inhibition of B16F10 cell migration and homing to the lung, along with suppression of cancer stemness (Figure 4).

### Spleen memory cells are responsible for Zfra-mediated cancer suppression

**Figure 6:**
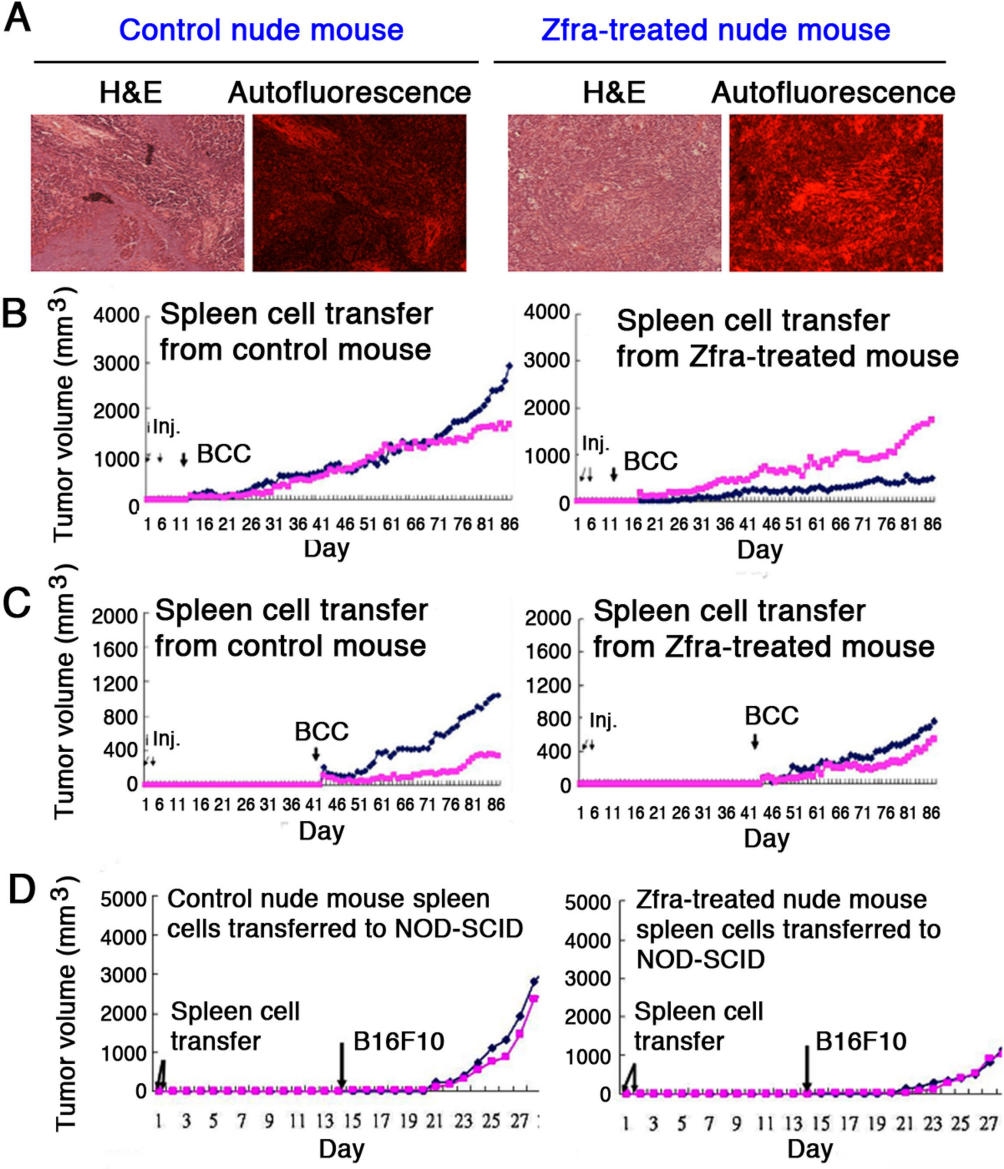
Zfra activates spleen cells for conferring cancer suppression **(A)** Nude mice were pre-injected with 100 μl of sterile MilliQ water or Zfra4–10 (4 mM in sterile water) via tail veins once per week for 3 consecutive weeks, and then inoculated with 2 million cells of melanoma B16F10 at both flanks of each mouse. Mice were sacrificed on day 40th, and spleens were isolated. Spleen microsections were prepared and stained with hematoxylin and eosin (H&E). Red autofluorescence was determined by a fluorescence microscope. **(B–C)** Nude mice were pretreated with Zfra4–10 (2 mM in 100 μl sterile water) or sterile water only via tail vein injections in 3 consecutive weeks. Spleen cells were isolated and injected into naïve nude mice (2 millions cells) through tail veins in 2 consecutive days. A week (panel B) or a month later (panel C), these mice were subcutaneously inoculated with BCC at both flanks. BCC growth was measured daily. **(D)** Similarly, nude mice were pretreated with Zfra4–10 (2 mM in 100 μl sterile water) or sterile water only via tail vein injections in 3 consecutive weeks. Spleen cells were isolated and injected into naïve NOD-SCID mice (2 millions cells) through tail veins in 2 consecutive days. Two weeks later, these mice were subcutaneously inoculated with melanoma B16F10 cells at both flanks. Tumor growth was measured daily. Spleen cells from Zfra4–10-treated nude mice conferred resistance to B16F10 growth in NOD-SCID mice. For above experiments, a representative data set is shown from 2 experiments.

Quite frequently, splenomegaly was observed in tumor-growing mice, and Zfra effectively suppressed the enlarged spleen by near 100% reduction. When Zfra1–31 and Zfra4–10 peptides were suspended in PBS, both peptides exhibited green and red autofluorescence under fluorescence microscopy (Supplementary Figure S4). However, when suspended in water, both Zfra peptides failed to exhibit autofluorescence (Supplementary Figure S4). When Zfra4–10 was delivered to mice via tail veins, the peptide became trapped predominantly in the spleens but not in other organs (Figure 6A; Supplementary Figure S5). Organs, including brain, kidney, liver, lung, small intestine, skin, and the B16F10 tumors, were negative for Zfra (Supplementary Figure S5A). No apparent damage to the liver was shown due to Zfra treatment (Supplementary Figure S5B). Zfra is not toxic to animals, and that Zfra-treated mice can live for their entire life span (data not shown). The observations suggest that Zfra induces memory spleen cells to block cancer growth.

To test the hypothesis that memory spleen cells participate in Zfra-mediated cancer suppression, spleen cells from control and Zfra-treated nude mice were isolated and transferred to naïve mice via tail veins. These mice were then allowed to rest for 1 week or 1 month, followed by inoculation with BCC. Naïve nude mice received Zfra-treated spleen cells became more resistant to the growth of BCC xenografts than those mice received control spleen cells (Figure 6B, 6C). The observations suggest the presence of Zfra-activated spleen cells in blocking cancer growth. Similar experiments were performed and shown to exhibit the acquired resistance to breast cancer cells in spleen cell transfer experiments in nude mice.

When T- and B-cell deficient NOD-SCID mice were pre-injected with Zfra4–10 (or sterile MilliQ water) for 3 consecutive weeks, these Zfra-treated mice did not resist the growth of inoculated B16F10 cells (Supplementary Figure S6). However, when NOD-SCID mice received Zfra-treated spleen cells from nude mice, these mice were able to resist the growth of B16F10 xenografts (Figure 6D).

### Isolation of novel Zfra-binding spleen lymphocytes and their dramatic down-regulation in tumor-bearing mice

**Figure 7:**
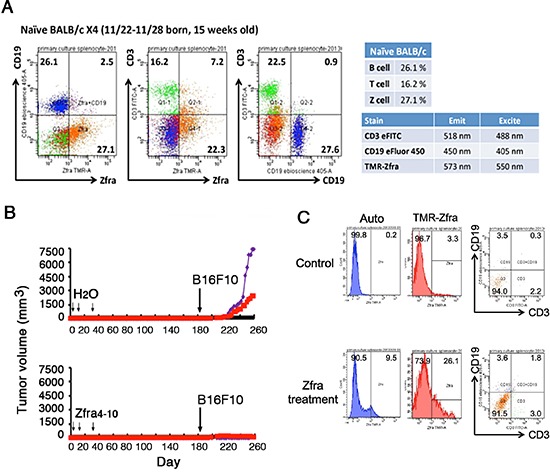
Down-regulation of spleen Zfra-binding Z cells in tumor-growing mice **(A)** Spleen lymphocytes were isolated from a 15-week-old BALB/c mouse, and stained using TMR-Zfra and specific fluorescent antibodies against CD3 and CD19 for T and B cells, respectively. **(B–C)** BALB/c mice were pretreated with Zfra4–10 (2 mM) and became resistant to the growth of inoculated B16F10 melanoma cells. By cell sorting, TMR-Zfra-positive Z spleen cells were around 25% in Zfra-treated mice. The isolated Z cells did not exhibit T and B cell markers, as further staining with CD3 and CD19 antibodies. In B16F10 melanoma-growing BALB/c mice, spleen Z cells dropped down to 3.3% or even lower. In untreated control mice, Z cells are around 25–29%. A representative data set is shown from 2 experiments.

We isolated Zfra-binding spleen cells by cell sorting. Zfra1–31 was labeled with tetramethylrodamine, designated TMR-Zfra. TMR-Zfra-positive spleen cells, tentatively named Z cells, were around 25–29% in the normal spleen of BALB/c mouse, as determined by cell sorting (Figure 7A). By using specific antibodies, the spleen T and B cell populations were also determined, which were 16 and 26%, respectively (Figure 7A). Overlaps between T and B or Z cells were 7% or less, suggesting that the Zfra-binding spleen cells could have a unique phenotype.

Again, we showed that Zfra4–10 blocked B16F10 growth in BALB/c mice (Figure 7B). These mice were sacrificed for determining the levels of spleen Z cells. B16F10-growing BALB/c mice had low levels of Z cells, which were 3.3% or even lower (Figure 7C). In contrast, Zfra-treated mice had 26.1% Z cells (Figure 7C), which is within the normal range in naïve mice. Importantly, Z cells do not exhibit the specific T and B cell markers, as determined using fluorescent CD3 and CD19 antibodies, respectively (Figure 7C). In a similar experiment, Zfra4–10 was shown to block the growth of neuroblastoma NB69 cells in nude mice (Supplementary Figure S7). The isolated spleen Z cell population was around 38.5% in the Zfra4–10-treated nude mice. The cells are devoid of T and B cell-specific markers CD27 and CD19, respectively (Supplementary Figure S7).

### Identification of Hyal-2+ CD3− CD19− Z cells and activating Z cells *in vitro* for suppressing cancer growth *in vivo*

**Figure 8:**
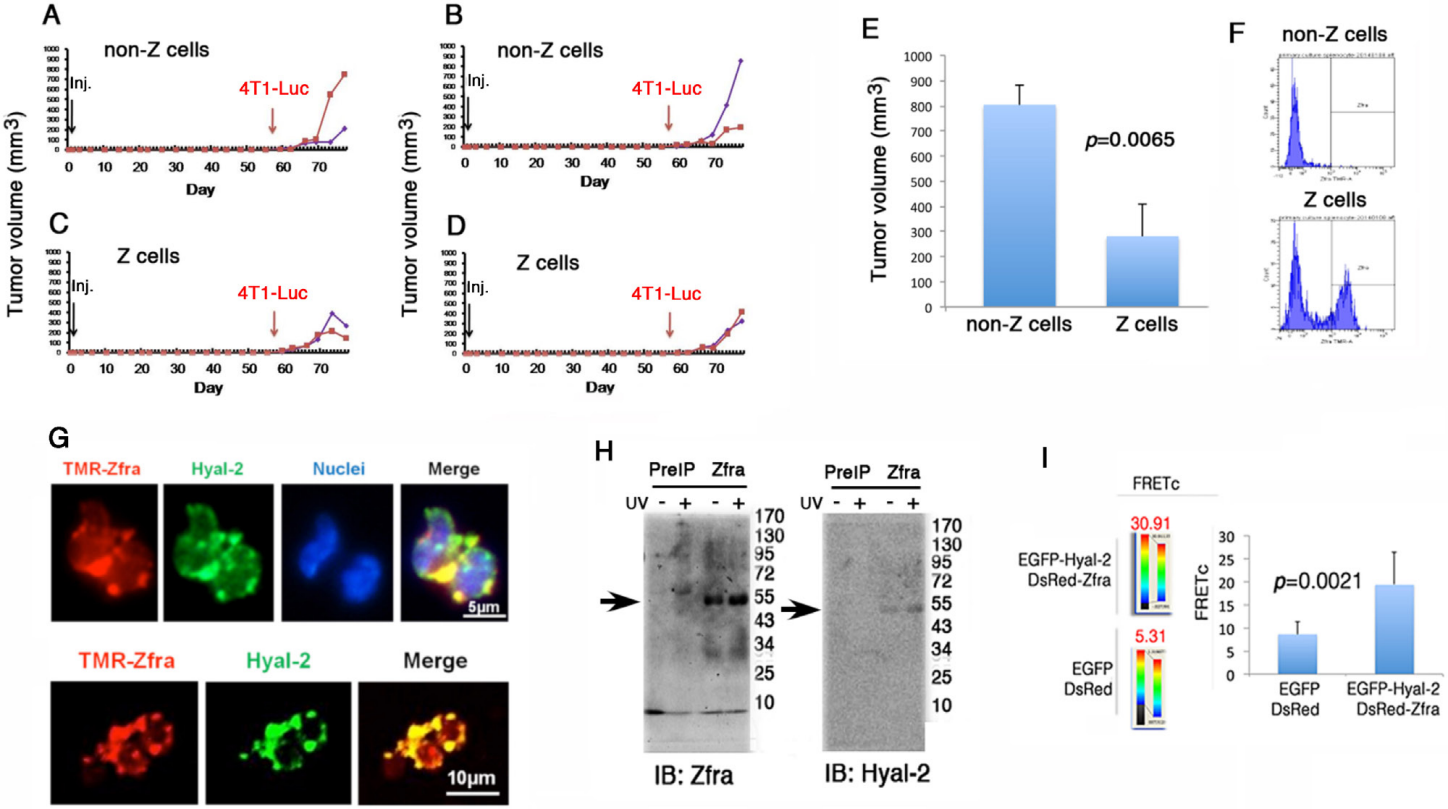
*In vitro* stimulation of Hyal-2+ CD3− CD19− Z cells for killing breast cancer *in vivo* **(A–F)** 6 naive BALB/c mice were sacrificed and spleen cells were isolated. These cells were treated with 20 μM Zfra4–10 for 16 hr. Z+ and Z-cells were isolated by cell sorting (see F). The isolated cells were then transferred to naïve BALB/c mice. Syngeneic breast cancer 4T1-Luc cells did not grow effectively in mice inoculated with Z+ cells (mean standard deviation; *n* = 6). **(G)** Mouse spleen cells were stained with TMR-Zfra (red) and antibody against Hyal-2 (green). Confocal analysis revealed the co-localization of both proteins. **(H)** Aliquots of full-length Zfra1–31 (100 μM) were added to the whole lysates of Hyal-2-positive L929 fibroblasts, treated with or without UV. Post incubation at room temperature for 30 min, the mixtures were subjected to centrifugation at 13,000 rpm in a microfuge for 15 min. The precipitates were analyzed by Western blotting for the co-presence of exogenous Zfra and endogenous Hyal-2. **(I)** COS7 fibroblasts were co-transfected with expression constructs of EGFP-Hyal-2 and DsRed-Zfra. FRET analysis revealed the binding of Hyal-2 with Zfra (see increased FRETc), whereas EGFP and DsRed failed to interact with each other.

We examined the possibility of activating Z cells *in vitro* for suppressing cancer growth *in vivo*. Spleen cells were isolated from 6 naïve BALB/c mice and then cultured overnight. These cells were stimulated with Zfra4–10 peptide (20 μM) for 16 hr, and then stained with TMR-Zfra for cell sorting. Z+ and Z– cell populations were counted and injected via tail veins of BALB/c. Post injection for 2 months, these mice were challenged with syngeneic breast cancer 4T1-Luc cells. Breast cancer cell growth was significantly suppressed in mice received with Z+ cells (Figure 8A–8F).

Next, we explored the nature of Z+ cells. Naïve mouse spleen cells were isolated and stained with TMR-Zfra (red) and antibody against membrane Hyal-2 (green) for confocal microscopy. The results showed the co-localization of both TMR-Zfra and Hyal-2 proteins on the cell surface (Figure 8G). Synthetic Zfra1–31 and Zfra4–10 are able to interact with cytosolic proteins in a covalent manner (Lee et al., unpublished). Hyal-2-positive L929 fibroblasts were treated with or without UV, and then incubated at 37^o^C for 30 min. Whole cell lysates were then prepared and mixed with Zfra1–31. The mixtures were centrifuged, and the precipitates were shown to possess the complex of exogenous Zfra and endogenous Hyal-2 (Figure 8H). Hyaluronidase Hyal-2 is a known cell surface protein [19–21]. By FRET (Förster resonance energy transfer) microscopy [13, 14], we further determined the binding of transiently overexpressed EGFP-Hyal-2 with DsRed-Zfra in COS7 fibroblasts (see increased FRETc) (Figure 8I). No EGFP and DsRed binding was shown in control cells (Figure 8I).

**Figure 9:**
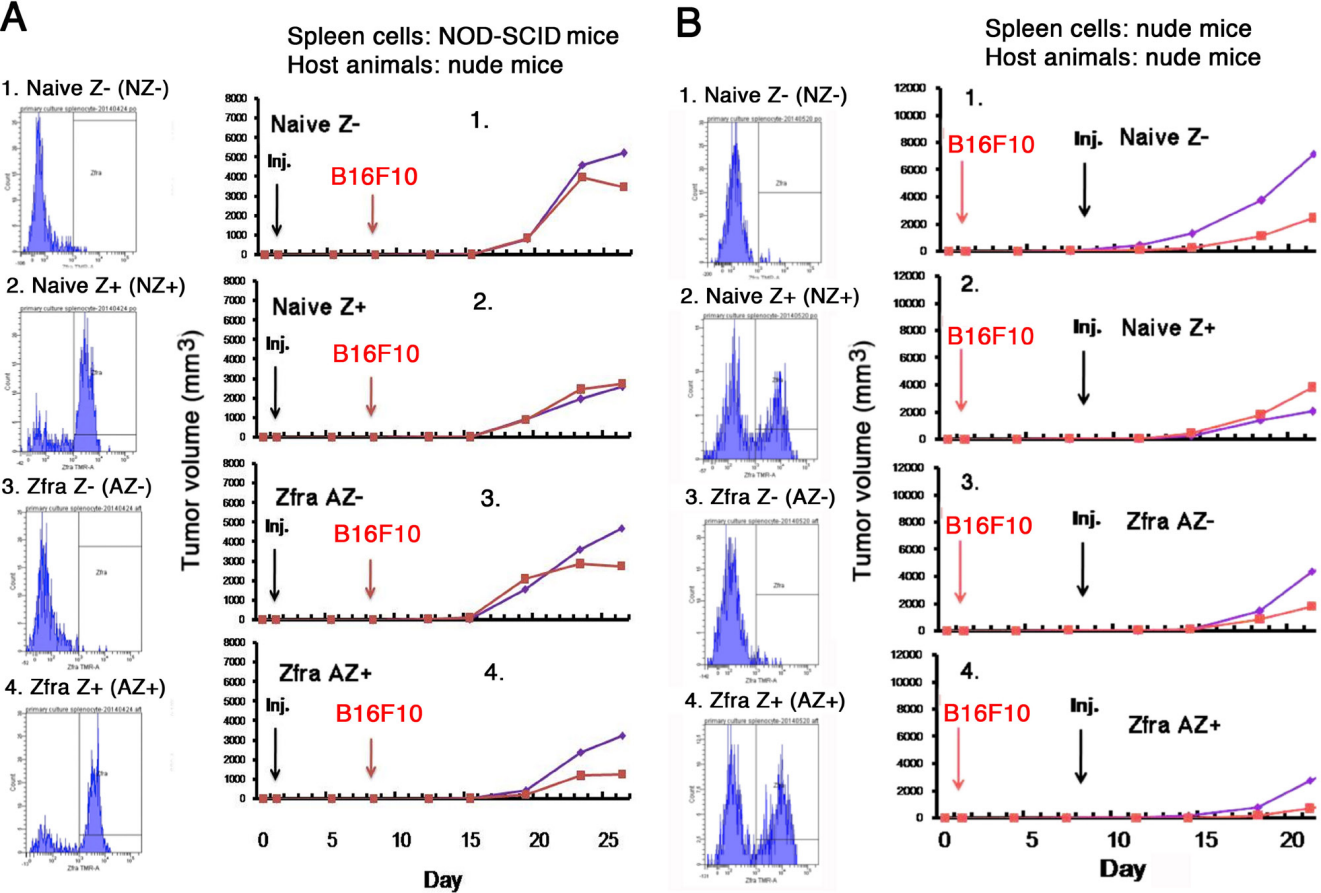
Activated Z cells inhibit melanoma growth *in vivo* **(A)** Spleen cells were isolated from 8 naïve non-T and non-B NOD-SCID mice, then treated with or without 20 μM Zfra4–10 for 24 hr in cell culture, and finally sorted using TMR-Zfra using a cell sorter. The resulting 4 types of cells, including naïve TMR-Zfra- (NZ−), naïve TMR-Zfra+ (NZ+), Zfra-activated TMR-Zfra- (AZ−), and Zfra-activated TMR-Zfra+ (AZ+), were injected to each indicated recipient naïve nude mouse via tail veins. One week later, B16F10 cells were inoculated into both flanks of each mouse. **(B)** Four naïve nude mice were inoculated with B1F10 cells on both flanks and allowed to rest for one week. Similar experimental steps were used to isolate and prepare the 4 populations of aforementioned spleen cells from nude mice. These cells were inoculated onto both flanks of the B16F10-growing nude mice. For both experiments, the sorting profiles for isolated cell types are shown. The kinetics of B1F10 cell growth on both flanks in mice is shown.

In similar experiments, spleen cells were isolated from naïve NOD-SCID mice, followed by stimulating with or without Zfra4–10 for 16 hr *in vitro*. Cells were then sorted using TMR-Zfra and injected to each recipient naïve nude mouse via tail veins. Growth of B16F10 cells in these mice revealed that Zfra-activated Z+ cells were effective in blocking melanoma growth (Figure 9A). Intriguingly, greater than 80% of spleen cells in the naïve NOD-SCID mice are TMR-Zfra positive (Figure 9A), as compared to those in BALB/c and nude mice (Figures 7 and 8B).

Additionally, 4 naïve nude mice were inoculated with B1F10 cells on both flanks, then allowed to rest for one week, and inoculated with Zfra-activated Z+ cells, as prepared using other naïve nude mice. Again, Zfra-activated Z+ cells were effective in blocking melanoma growth (Figure 9B).

## DISCUSSION

**Figure 10:**
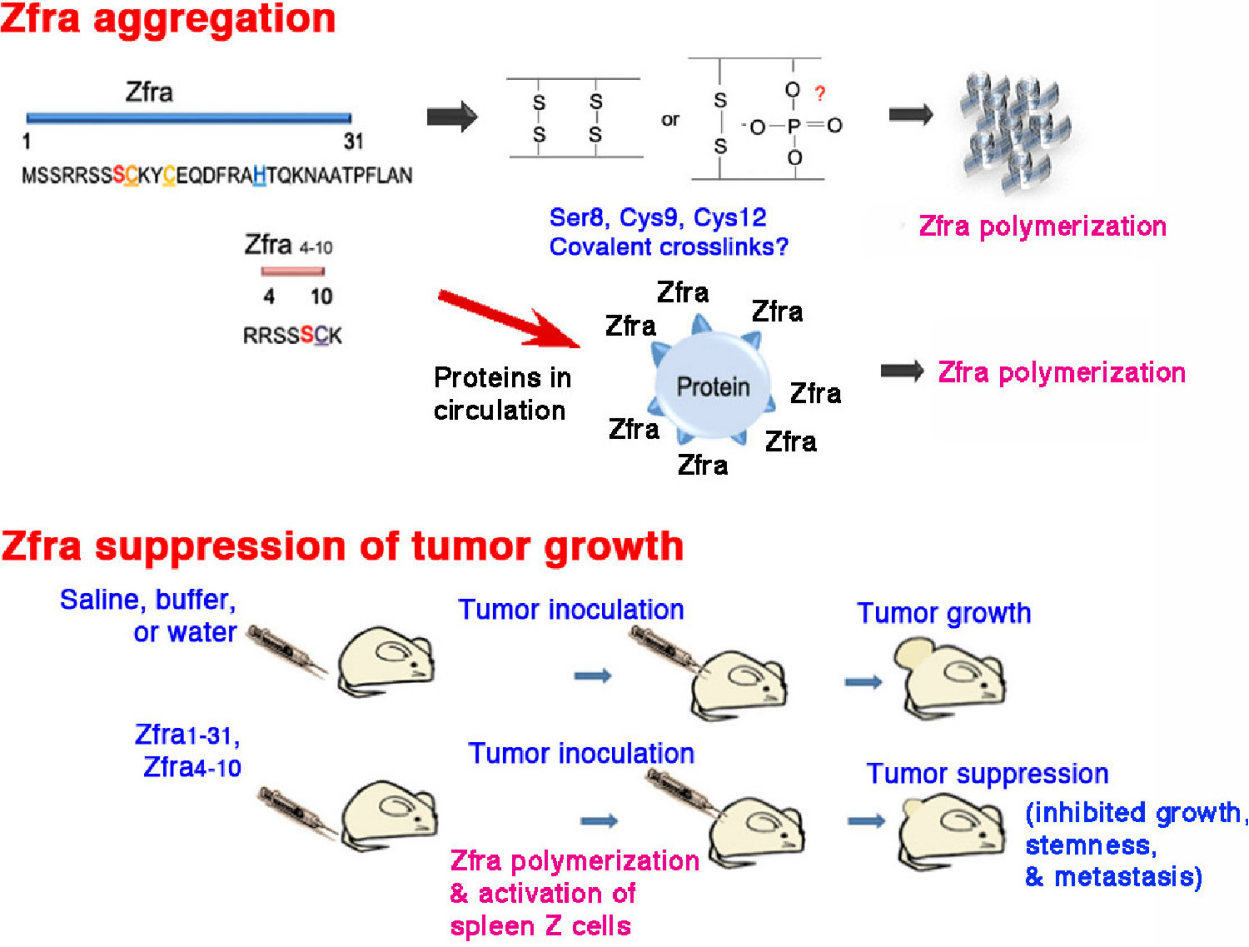
A schematic graph of Zfra polymerization and anticancer function **(A)** Zfra self-polymerization *in vitro*. Potential crosslinking bonds are shown. Binding of injected Zfra with proteins in circulation is proposed. **(B)** The anticancer function of Zfra *in vivo*.

In summary, highly purified synthetic Zfra tends to undergo self-polymerization in enzyme-free PBS, and this correlates with its anticancer activity *in vivo* (Figure 10). Both Cys9 and Cys12 are potential amino acids that are involved in peptide self-polymerization. Since the ladder-like polymerization pattern is similar under both non-reducing and reducing SDS-PAGE, the observation suggests that cysteines do not contribute to the formation of the polymerization pattern and raises the possibility of other amino acids, which are involved in the crosslinking (Figure 10). Ser8 is a candidate amino acid residue. Alteration of Ser8 to Gly8 abolishes the self-polymerization. Additional data revealed that both Zfra1–31 and Zfra4–10 undergo self-polymerization in a covalent manner in the absence of catalytic enzymes (Lee *et al*., unpublished).

When immune-deficient nude mice and immune-competent BALB/c mice received the full-length Zfra1–31 or truncated Zfra4–10 via tail veins, these mice became resistant to the growth, metastasis and stemness of melanoma cells and many other malignant cancer cells (Figure 10). We have shown that Zfra significantly suppresses the expression of stemness markers Oct-4 and SSEA-4, along with dramatically reduced TIAF1 expression, in the lung of mice inoculated with melanoma B16F10. In comparison, TIAF1 appears to become fibrous in the melanoma lesions in the lung of control mice. Fibrous TIAF1 in the cancer lesions probably supports cancer progression, although monomeric TIAF1 acts synergistically with tumor suppressors WWOX and p53 to block cancer growth [13–15]. Downregulation of WWOX is known to induce aggregation of TIAF1 and TRAPPC6A (Trafficking Protein Particle Complex 6A) in the brain cortical areas of mice and humans [18, 22].

Zfra also cured the spontaneously developed tumors in the heterozygous *Wwox* +/− mice. Zfra became polymerized in the circulation and was mainly deposited or filtered in the spleen due to its autofluorescence. The observations suggest that the anticancer function of Zfra is via spleen cells. Both Zfra1–31 and Zfra4–10 peptides were freshly prepared in sterile water or PBS. Overly polymerized Zfra, caused by high concentrations or excessive salt *in vitro*, may abolish its anticancer function *in vivo* (data not shown). The notion that spontaneous tumor formation in WWOX +/− mice needs further investigation, since the wild type mice tend to spontaneously grow tumors. Our findings are in contrast to a previous report [23].

We determined for the first time that Zfra-binding Hyal-2+ CD3− CD19− Z spleen cells are responsive to Zfra stimulation for mounting against the growth of a broad spectrum of cancer cells *in vivo*, suggesting that a therapeutic approach can be developed using patients' own Z cells for cancer treatment. Zfra activates or educates memory Z cells *in vitro* from the isolated naïve spleen cells of both immune deficient and competent mice. Once transfer of these purified memory Z cells into another naïve mice, an anticancer memory response is effectively installed. Traditionally, immune deficient mice are utilized for growing cancer xenografts, as they fail to elicit an immune response to run against the cancer growth. Clearly, the Hyal-2+ CD3− CD19− Z cell lineage is an effector memory system, which can be educated for eradicating cancer cells. How these cells fight against cancer cells is being determined.

The genetic signature of Z cells, which are non-T and non-B, has yet to be determined. The levels of Z cells are very low or near 0% in tumor-growing mice, suggesting that growing tumor cells may eradicate the uneducated or naïve Z cells *in vivo*, thereby ultimately costing lives. Accordingly, the best strategy to overcome cancer is to educate or activate autologous Z cells *in vitro* for suppressing cancer growth *in vivo*.

The polymerized Zfra binds membrane Hyal-2 and this may induce the signaling of Hyal-2, WWOX and Samd4 [18–20 and unpublished]. No apparent internalization of Zfra is shown in spleen cells *in vitro* and *in vivo*. That is, there is a prolonged retention of Zfra on the cell surface to instigate and then desensitize the signaling. Since Hyal-2 is a co-receptor with CD44 for hyaluronan [18], we were able to reproduce the Zfra effect using anti-Hyal-2 IgG and high affinity hyaluronan for Hyal-2 (Su et al., unpublished). NOD-SCID mice do not have functional T, B and NK cells. By cell sorting, we showed that there are greater than 80% of spleen cells, which are TMR-Zfra positive or Z+ in the NOD-SCID mice. The naïve cells, with or without Zfra stimulation, do not have the anticancer activities upon transferring to another naïve NOD-SCID mice (data not shown). However, Zfra induces the anticancer activities of Z+ cells from NOD-SCID mice in less than 24 hr *in vitro*, and these activated Z+ cells become tumor suppressing in other mouse strains. Indeed, NOD-SCID mice are refractory to Zfra induction for the anticancer response. Mechanism of this regard is being determined.

It is generally believed that antigen-presenting cells (APC) are needed to present antigens to T cells for clonal activation [24]. Whether antigen-presenting cells (APC) are involved in processing polymerized Zfra for presenting to Z cells is not known and remains to be determined. Z cells do not appear to be associated with memory NK cells, as these cells mainly reside in the liver [25, 26]. Zfra is not a cancer antigen. Indeed, polymerized Zfra may undergo extensive crosslinks, as shown by mass spectrometry analysis (Lee et al., unpublished). Whether these neoantigens are similar to those on cancer cell surface remains to be determined.

The outstanding feature of Zfra is its capability in strongly suppressing the stemness and metastasis of cancer cells [27, 28]. As shown using melanoma B16F10 cells, Zfra blocks the expression of Oct4 and SSEA-4 in these cancer cells in the mouse lung. Zfra did not inhibit the growth of glioma 13–06-MG cells. However, Zfra completely blocked the metastasis of 13–06-MG cells to the lung. Cancer patients frequently die from cancer cell metastasis. Control of cancer cell metastasis by Zfra is critical for survival of cancer patients. Whether activated Z cells are present in the cancer lesions that result in the suppression of cancer metastasis remains to be established.

Failure of Zfra in the suppression of the growth of 13–06-MG cells is probably due to insufficient amounts of Zfra used. Indeed, insufficient doses of Zfra may lead to increased tumor growth. Normally, mice receive 1–4 mM or 70–500 μg of Zfra (in 100 μl sterile water) via tail vein injections. The concentrations of Zfra are diluted *in vivo* and not toxic to the animals, as Zfra-treated mice can survive for an entire life span around 2–2.5 years. Direct injection of Zfra into growing breast MDA-MB-435s cells in nude mice could result in suppression of cancer cell growth. Zfra also cures tumors spontaneously developed in *Wwox* gene heterozygous mice (70% cure of test mice). *In vitro* experiments, isolated spleen cells are treated with 10 or 20 μM of Zfra. Indeed, this is sufficient in inducing the anticancer response in Z+ spleen cells once transferred back *in vivo*.

Taken together, our data thus far have clearly indicated that Zfra can initiate an effective anticancer response via the novel Hyal-2+ CD3− CD19− immune cell lineage. We have successfully blocked the growth of many malignant cancers *in vivo*, including melanoma, skin basal cell carcinoma, neuroblastoma, breast adenocarcinoma, prostate adenocarcinoma, lung adenocarcinoma, and others. Certain glioblastoma cells are sensitive to Zfra-mediated growth suppression, whereas some are not. Nonetheless, we have established a feasible approach in both cancer prevention and treatment. The duration of anticancer memory response in activated Z cells needs to be established. Hopefully, the ultimate goal of our study is to achieve a “one shot strategy” for lifetime immunization against all types of cancers.

## MATERIALS AND METHODS

### Peptides

Zfra and other indicated peptides were synthesized manually by the Genemed Synthesis (San Antonio, Texas, USA). Manual synthesis was used to prevent peptide oxidation. Automatic synthesis may cause inactivation of Zfra peptides in their anticancer functions. These peptides are: 1) Zfra1–31, NH-MSSRR SSSCKYCEQDFRAHTQKNAATPFLAN-COOH; 2) Zfra1–15, NH-MSSRRSSSCKYCEQD-COOH; 3) Zfra16–31, NH-FRAHTQKNAATPFLAN-COOH; 4) Zfra4–10, NH-RRSSSCK-COOH; 5) Zfra5–10, NH-RSSSCK-COOH; 6) Zfra1–15(S8G), NH-MSSRRSSGCKYCEQD-COOH; 7) Zfra4–10(pS8), NH-RRSS**pS**CK-COOH (Ser8 phosphorylation); 8) Zfra5–10(ΔC9), NH-RSSSK-COOH (Cys9 deletion); 9) Zfra5–10(ΔC9/pS8), NH-RS**pS**K-COOH (Ser8 phosphorylation and Cys9 deletion); 10) WWOX28–42, NH-KDGWVYYANHTEEKT-COOH (used in control experiments); 11) TMR-Zfra, Zfra1–31 or Zfra4–10 was labeled with a red-fluorescent Texas Red maleimide fluorescent probe tetramethylrodamine (Genemed Synthesis). The peptide stocks were made as 10 mM in degassed sterile MilliQ water. Each tube was flushed with nitrogen and stored in –80°C freezer. For tail vein injections, peptides were prepared fresh in degassed MilliQ water at 1–4 mM in 100 μl MilliQ.

### Animals

6–8 week old male BALB/c mice, NOD-SCID (NOD.CB17-*Prkdc^scid^*/NCrCrl) mice (Laboratory Animal Center, NCKU) or nude (BALB/cAnN.Cg-*Foxn1^nu^*/CrlNarl) mice (National Laboratory Animal Center, Taiwan) were used. For experimental designs, we followed the approved protocols from the Intramural Animal Use and Care Committee (IACUC) of the National Cheng Kung University. Mice were intravenously injected into tail veins with an aliquot of Zfra_1–31_, Zfra_4–10_ or indicated peptides (10–40 mM in 100 μl sterile MilliQ water or sterile PBS). Post injection for 1–8 weeks, cancer cells (2–2.5 × 10^6^ cells/100 μl saline) were then inoculated in subcutaneous sites at both flanks. Tumor volumes were measured daily and calculated using the equation: *D* x (*d*)^2^ /2, where *D* and *d* are the major and minor diameters, respectively. Cancer cell lines used in the experiments were: 1) human malignant breast MDA-MB-231, MDA-MB-435s, and MDA-MB-468; 2) human malignant prostate DU145; 3) human epithelial virus-transformed prostate PZ-HPV-7; 4) human lung NCI-H1299; 5) human neuroblastoma SH-SY5Y and NB69; 6) human malignant glioma U-87-MG and 13–06-MG and mouse glioma CNS-1; 7) human skin basal cell carcinoma BCC; 8) mouse melanoma B16F10; 9) Hyal-2-expressing L929 fibroblasts [18, 19]. The mouse glioma CNS-1 and human U87-MG and 13–06-MG cells were provided by Dr. Chun-I Sze of the Department of Anatomy and Cell Biology, National Cheng Kung University (NCKU). Prostate PZ-HPV-7 cells were from Dr. Pei-Jung Lu, Institute of Clinical Medicine, NCKU, and NB69 cells from Dr. Christina L. Chang, Institute of Molecular Medicine, NCKU.

### Tissue sections and immunohistochemistry

Tissue sections and immunohistochemistry were carried out as described [13, 14]. Antibodies against TIAF1 were used as described previously [13, 14].

### Cell staining and sorting analysis

Mice were sacrificed and spleen cells were collected. Synthetic peptide TMR (Tetramethylrhodamine)-Zfra1–31 was made (Genemed Synthesis; excitation 550 nm, emission 573 nm). Specific antibodies for spleen cell staining were from eBioscience, including CD3e FITC (excitation 488 nm, emission 518 nm), CD27 FITC (excitation 488 nm, emission 518 nm), CD19 eFluor 450 (excitation 405 nm, emission 450 nm), mouse CD19 PerCP-Cyanine5.5 (excitation 488 nm), and mouse F4/80 antigen FITC (excitation 488 nm). Flow cytometry (FACSCalibur BD) analysis and cell sorting (FACSAria BD) were performed to analyze spleen cell populations. Data were analyzed by the WinMDI 2.8 program.

### Data presentation and statistical analysis

A representative data from the kinetics of tumor cell growth in tumor-bearing mice is shown from 2–6 experiments, as specified. Where indicated, end-point data for tumor growth were analyzed by Student's *t* test.

## SUPPLEMENTARY FIGURES


