# Supplementary material for: Zfra activates memory Hyal-2+ CD3− CD19− spleen cells to block cancer growth, stemness, and metastasis in vivo
Source: Oncotarget. 2015 Feb 19;6(6):3737–51. doi: 10.18632/oncotarget.2895 (PMC4414150; doi:10.18632/oncotarget.2895)
Supplement: Supplementary file 1 [file oncotarget-06-3737-s001.pdf]

SUPPLEMENTARY FIGURES

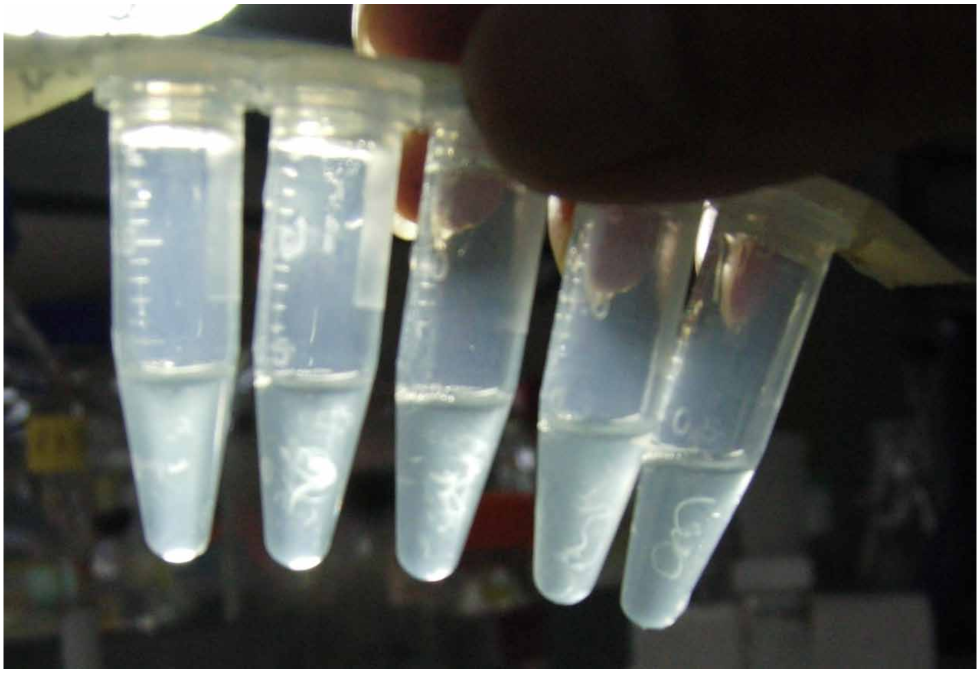

Supplementary Figure S1: Zfra precipitates in phosphate-buffered saline (PBS). Synthetic full length Zfra peptide (>90% pure; 5 mM) was resuspended in degassed PBS. Zfra formed long fibrous visible precipitates in less than 5 hr in room temperature (5 repeats).

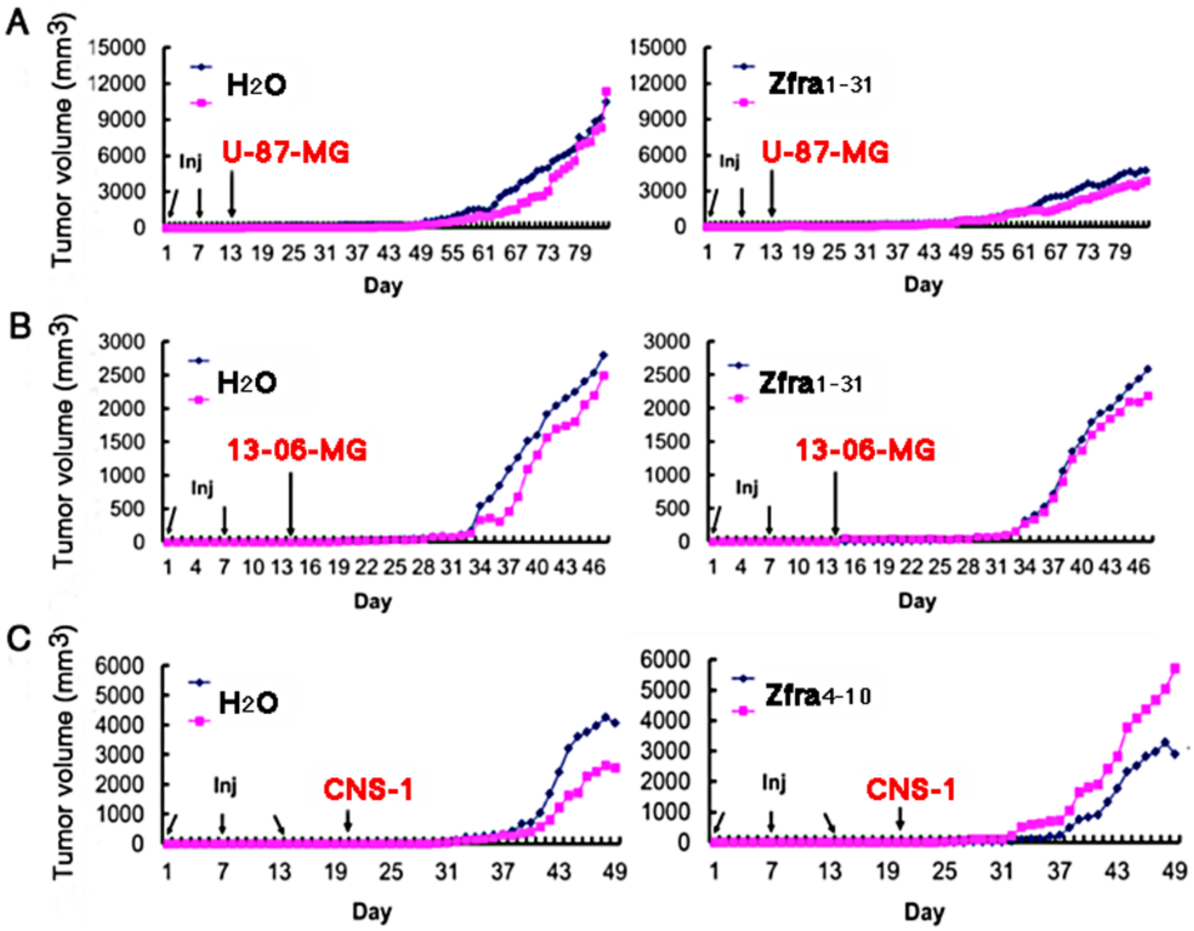

Supplementary Figure S2: Zfra suppresses the growth of glioma U-87-MG cells. **(A–C)** Nude mice were pre-injected with sterile MilliQ water, Zfra4-10, or Zfra1-31 (3 mM in 100 µl sterile water) in 2 or 3 consecutive weeks, followed by inoculating 2 million cells of glioma U-87-MG (human), 13-06-MG (human), or CNS-1 (mouse) one week later. Please note that A is the same as in Figure 1C.

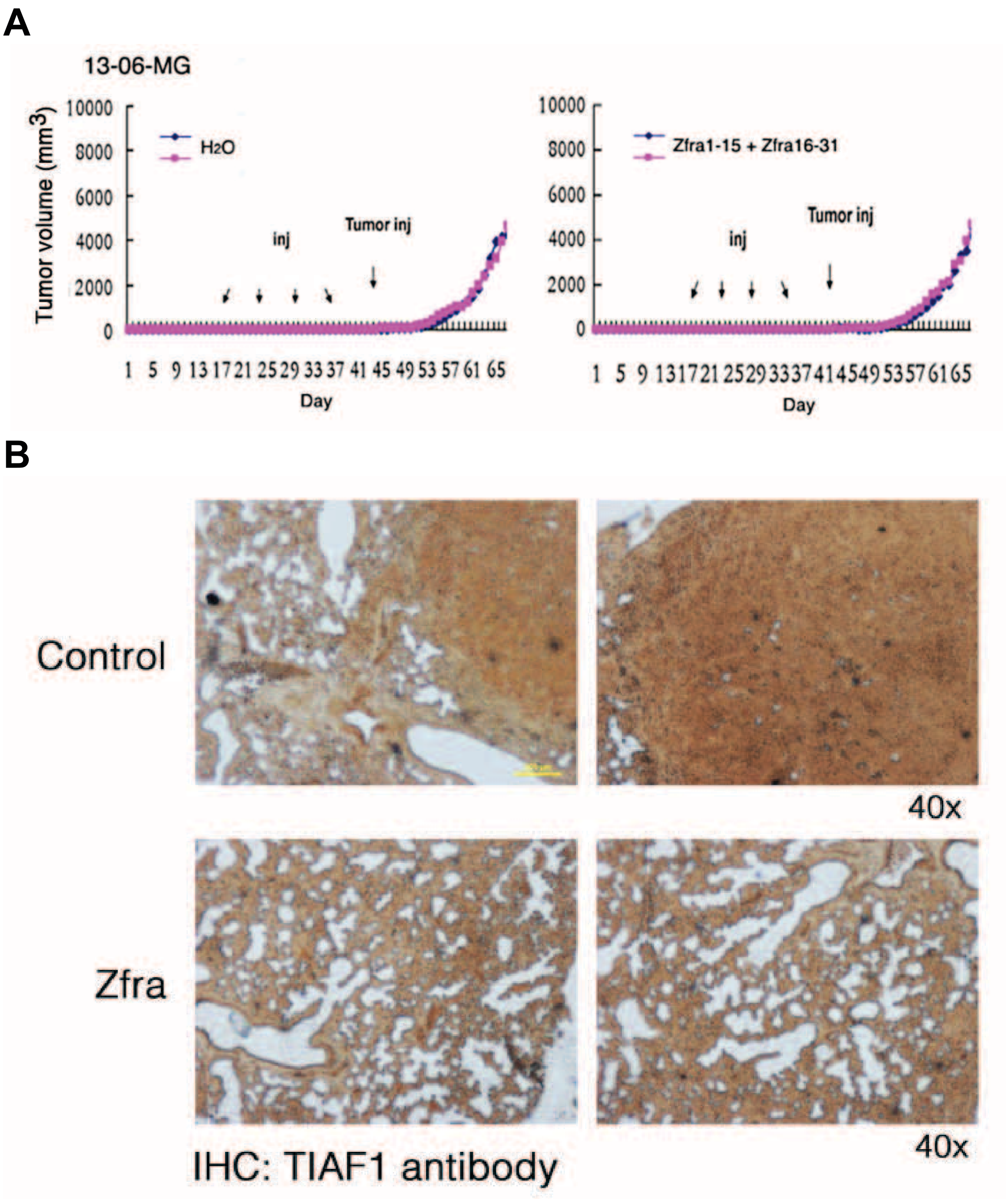

Supplementary Figure S3: Zfra1-15 and Zfra16-31 in combination did not block the growth of glioma 13-06-MG cell growth but prevented cancer metastasis to the lung. (A) Nude mice were pre-injected with sterile MilliQ water or Zfra1-15 and Zfra16-31 in combination (4 mM in 100 µl sterile water) for 4 consecutive weeks, and then inoculated 2 million cells of glioma 13-06-MG at both flanks of each mouse. **(B)** Metastasis of 13-06-MG cells to the lung was blocked by Zfra1-15 and Zfra16-31. TIAF1 is shown in the lung cancer lesion (IHC). Two representative lung sections are shown from control and Zfra-treated mice. Scale bar: 200 µM.

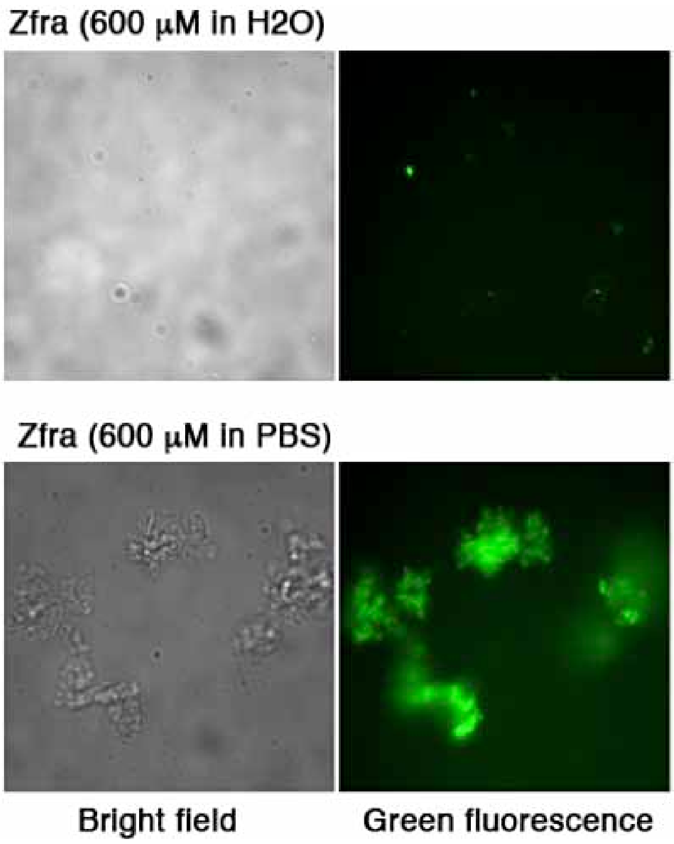

Supplementary Figure S4: Zfra exhibits autofluorescence upon polymerization. The full-length Zfra were dissolved in MilliQ water or PBS. Zfra became polymerized in PBS but not in water. Green or red autofluorescence was shown when Zfra was in PBS but not in water (data not shown for red fluorescence). Similar results were observed for Zfra4-10 (data not shown).

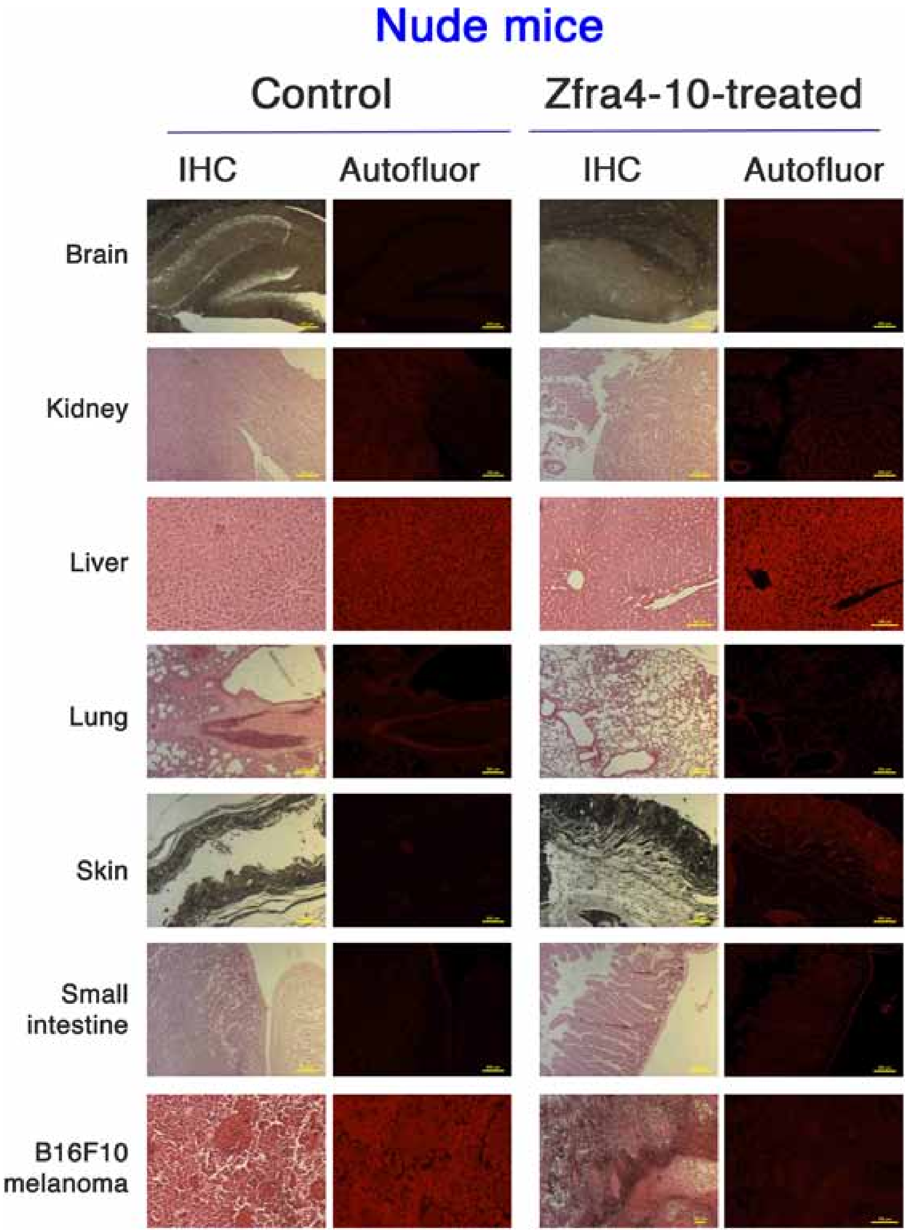

Supplementary Figure S5: Injected Zfra is deposited in the spleen. **(A)** Nude mice were pre-injected with 100 µl of sterile MilliQ water or Zfra4-10 (4 mM in sterile water) via tail veins for 3 consecutive weeks, and then inoculated with 2 million cells of melanoma B16F10 at both flanks of each mouse. Upon sacrifice of the mice on the day 40th, spleens and indicated organs were harvested and fixed, and tissue sections were prepared and stained with hematoxylin and eosin (H&E). Autofluorescence (Autofluor) of Zfra was examined by microscopy. Scale bar in A is 200 µm (40x magnification). (*Continued*)

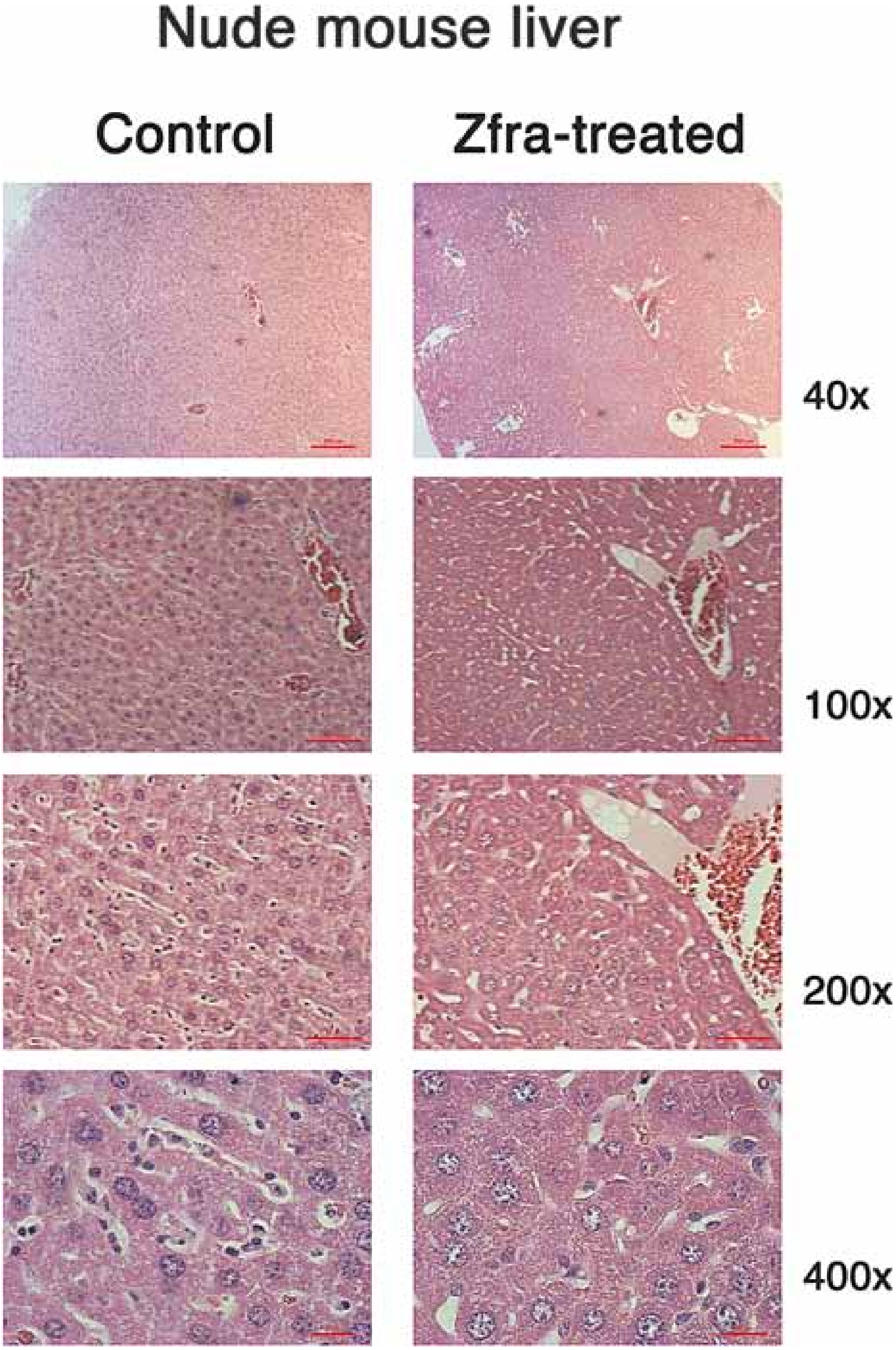

Supplementary Figure S5: (*Continued*) (B) Liver sections were stained by H&E.

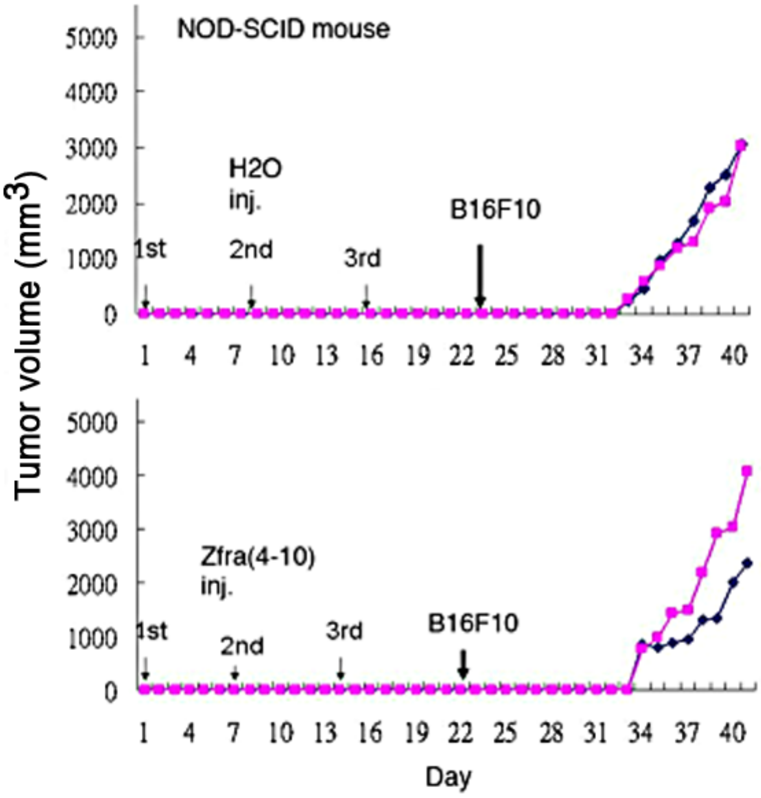

Supplementary Figure S6: Zfra fails to block melanoma growth in NOD-SCID mice. NOD-SCID mice were pre-injected with 100 µl of sterile water or Zfra4-10 (2 mM in sterile water; see arrows) once per week in 3 consecutive weeks. Mice were then inoculated with B16F10 cells. Zfra4-10 did not block B16F10 growth in NOD-SCID mice. A representative data set is shown from 3 experiments.

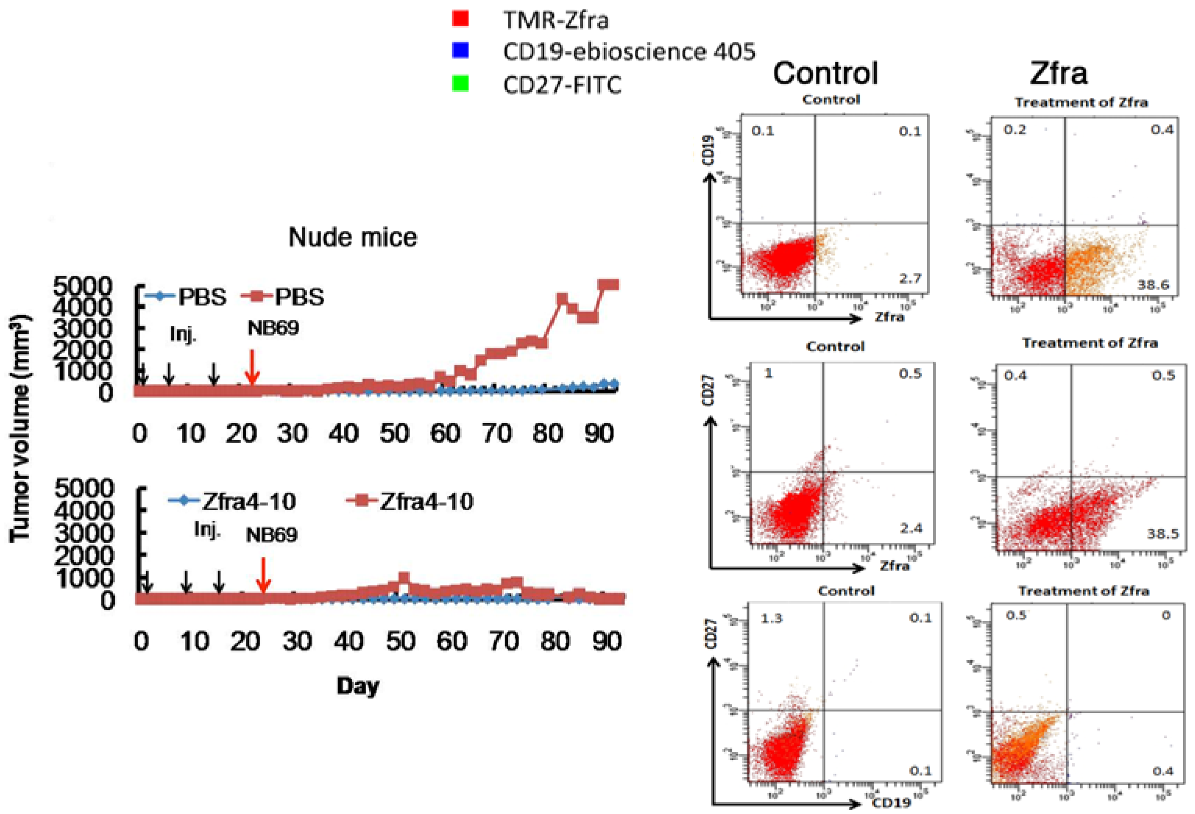

Supplementary Figure S7: Zfra blocks the growth of neuroblastoma NB69 cells in nude mice. Nude mice were pretreated with Zfra4-10 (2 mM in PBS) and resisted the growth of inoculated NB69 cells. By cell sorting, TMR-Zfra-positive Z spleen cells were around 38.5% in Zfra-treated mice. The isolated Z cells did not exhibit T and B cell markers, as further staining with CD27 and CD19 antibodies. In NB69-growing control nude mice, spleen Z cells dropped down to ~2.7% and lower. A representative data set is shown from 2 experiments.
